# Niemann-Pick disease type C clinical database: cognitive and coordination deficits are early disease indicators

**DOI:** 10.1186/1750-1172-8-35

**Published:** 2013-02-22

**Authors:** Miriam Stampfer, Susanne Theiss, Yasmina Amraoui, Xuntian Jiang, Sigrid Keller, Daniel S Ory, Eugen Mengel, Christine Fischer, Heiko Runz

**Affiliations:** 1 Institute of Human Genetics, University of Heidelberg, INF 366, Heidelberg 69120, Germany; 2 Center for Pediatric and Adolescent Medicine, University of Mainz, Mainz, Germany; 3 Diabetic Cardiovascular Disease Center, Washington University School of Medicine, St. Louis, MO, USA; 4 Molecular Medicine Partnership Unit (MMPU), University of Heidelberg/EMBL, Heidelberg, Germany

**Keywords:** Cholesterol, Clinical repository, Genotype-phenotype, Neurodegenerative disease, Oxysterols

## Abstract

**Background:**

The neurodegenerative lysosomal storage disorder Niemann-Pick disease type C (NP-C) is characterized by a broad clinical variability involving neurological, psychiatric and systemic signs. Diverse patterns of disease manifestation and progression considerably delay its diagnosis. Here we introduce the NP-C clinical database (NPC-cdb) to systematically obtain, store and analyze diagnostic and clinical findings in patients with NP-C. We apply NPC-cdb to study NP-C temporal expression in a large German-Swiss patient cohort.

**Methods:**

Current and past medical history was systematically acquired from 42 patients using tailored questionnaires. Manifestation of 72 distinct neuropsychiatric signs was modeled over the course of disease. The sequence of disease progression was re-constructed by a novel clinical outcome scale (NPC-cdb score).

**Results:**

The efficiency of current clinical diagnostic standards negatively correlates with duration of disease (p<3.9x10^-4^), suggesting insufficient sensitivity in patients early in the disease process. Neurological signs considered as typical for NP-C were frequent (e.g., cognitive impairment 86%, ataxia 79%, vertical supranuclear gaze palsy 76%) and their presence co-occurred with accelerated diagnosis. However, less specific neuropsychiatric signs were reported to arise considerably more early in the disease process (e.g., clumsiness -4.9±1.1 y before diagnosis). Most patients showed a steady disease progression that correlated with age at neurological onset. However, a distinct subcohort (n=6) with initially steadily progressing disease later showed a 2.9-fold accelerated progression that was associated with the onset of seizures (p<7x10^-4^), suggesting seizures as predictive for a poor prognosis.

**Conclusions:**

Considering early, but less specific neuropsychiatric signs may accelerate the path to diagnosing NP-C in a patient.

## Background

Niemann-Pick disease type C (NP-C; OMIM#257220; OMIM#607625) is a progressive lysosomal storage disorder with an estimated incidence of 1:120,000 live births [1-4]. Autosomal-recessive inheritance of mutations in either of two genes, *NPC1* and *NPC2,* are known causes [5,6]. Clinical features of NP-C encompass a wide spectrum of systemic, neurological and psychiatric signs. Systemic disease involves (hepato)splenomegaly and a risk for liver failure in affected infants. Characteristic neurological manifestations of NP-C are unrelated to visceral disease and include oculo-motor abnormalities (impaired saccadic eye movements, vertical supranuclear gaze palsy (VSGP)), cerebellar signs (ataxia, dystonia/dysmetria, dysarthria and dysphagia) and gelastic cataplexy. Upon disease progression, any sort of epileptic seizure may occur. Most patients develop cognitive impairment progressing to dementia. Schizophrenia-like psychosis is common in adults. Additionally, NP-C patients may show a multitude of less frequent and less specific symptoms [1,3,4].

The variable clinical picture, manifestation at different ages and diverse patterns of disease progression may delay the diagnosis NP-C for several years. The advent of plasma oxysterols as novel NP-C screening markers may help to partially reduce this diagnostic delay [7]. However, a comprehensive clinical assessment is considered as key to unambiguously assign the diagnosis [8]. An early diagnosis thus necessitates a better understanding of which clinical signs occur particularly early and at which sequence in the course of disease. This is particularly true for neurological symptoms as the major determinant of disease progression [4] and response to treatment [8-10]. To date, several studies have addressed how NP-C develops over time [11-18]. However, only rarely the disease spectrum has been analyzed comprehensively. Moreover, due to the rarity of NP-C, individual studies typically miss the statistical power to allow for generalization of their respective findings.

Here we have generated infrastructure to systematically assess, store and share NP-C specific clinical datasets as a resource for future large-scale collaborative studies. We apply our tools for a pilot study assessing the expression of NP-C in a large German-Swiss patient cohort. We identify distinct patterns of disease progression that correlate with specific neurologic manifestations. Moreover, our data suggest that an early diagnosis will profit from respecting less specific neuropsychiatric signs of NP-C.

## Methods

### Participants

**Figure 1:**
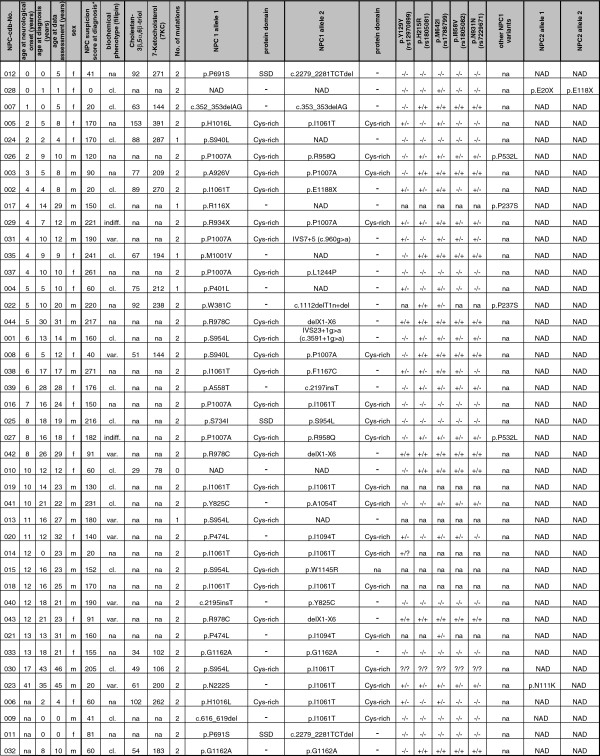
Diagnostic findings in studied NP-C patient cohort (n=42).* NP-C Suspicion Index (acc. to Wijburg et al., 2012): <40, NP-C unlikely; 40-69, NP-C follow up advised; ≥70, NP-C likely; na, *not available*; NAD, *nothing abnormal detected;* cl., *classic;* var., *variant;* indiff., *indifferent;* Cys-rich*, cystein-rich loop (aa 855-1098);* SSD*, sterol sensing domain (aa 615-797),* normal range: 3β,5α,6β-triol: 29-152; 7-KC: 78-390.

This is a two-center cross-sectional analysis of patient data. Data were acquired on 42 NP-C patients resident in Germany or Switzerland that (i.) at start of the study were still alive and (ii.) had been diagnosed within the past six years and/or long-term comprehensive medical records were available. Diagnosis NP-C was assured by sequencing of *NPC1* (NM_000271) and *NPC2* (NM_006432) genes, complemented by filipin staining of cultured skin fibroblasts as described [19,20] (Figure 1). Haplotypes of all patients (including six novel *NPC1* variants: c.1112delT1n+del, p.W381C, p.A558T, c.2197_2198insT, p.M1001V and p.F1167C) were entered into the online NP-C gene variation database (NPC-db; [21]). Data were assessed and evaluated by two clinical experts. Patients were diagnosed and/or followed-up at the Institute of Human Genetics Heidelberg, Germany and the Center for Lysosomal Storage Disorders Mainz, Germany.

### Standard protocol approvals, registrations, and patient consent

All subjects were enrolled with either their own or their representatives signed consent as covered by the IRB approval S-032/2012 from Heidelberg University. The study protocol conformed to the ethical guidelines of the 1975 Declaration of Helsinki.

### Data collection and assessment

Data were acquired over a period of 30 months (2009-2011) from personal interviews with the families and own or externally contributed longitudinal medical records. Neurological status of each patient at the time of the interview was assessed by clinical examination. Interview data were obtained with a customized questionnaire consisting of 270 questions on medical history, including 44 on diagnosis (Additional file 1). This questionnaire assisted to acquire data in a standardized format that allowed for systematic comparisons between patients. Data were then transferred as fixed terms and/or quantifiable parameters into an electronic Microsoft Office Access®-based database termed NP-C clinical database (NPC-cdb) (Additional file 2).

### Statistical analysis

For each of the 72 neuropsychiatric symptoms assessed, time from onset of neurological disease to occurrence of the respective symptom was documented. For patients not showing a respective symptom, time until the last visit was used as censored observation. Mean/median (incl. 95% confidence interval, 95%CI) timespan until occurrence was estimated by non-parametric censored Kaplan-Meier survival analysis using SPSS V20 (IBM Corp. Armonk, NY, USA). Fisher’s exact test was used to test for statistical significance of categorical data. Based on published data [8] sensitivity of the NP-C Suspicion Index tool was estimated as 95%. For association analyses of disease progression with seizures, relative NPC-cdb scores (cleaned from seizure-associated score points) two years before and after the first seizure event were determined and compared to disease progression in the cohort where no seizures had yet occurred.

### NPC-cdb score

NPC-cdb aims to document the comprehensive past and present clinical information available per patient. Based on our own experience and previous estimates [8,13,17], each symptom was subjectively scored according to its relative contribution to the general impairment of a patient (for guidelines on how to evaluate individual symptoms using the NPC-cdb score, see Additional file 3). A composite score (NPC-cdb score) was generated by adding up all past and current scores with each symptom contributing as a summand. Longitudinal scores were generated by reconstructing past medical history from interview results and clinical records. Reliability of the NPC-cdb score to adequately reflect NP-C severity was assured by assessing disease progression in all our patients also with the NPC clinical severity scale [17] (excluding evaluation of hearing, since required tests had been conducted only in few patients).

### Correlation of NPC-cdb score with plasma oxysterol levels

16 individuals of our cohort had been analyzed by Jiang et al., 2011 [22] for plasma levels of cholestane-3β,5α,6β-triol (3β,5α,6β-triol) and 7-ketocholesterol (7-KC) (Figure 1). For all patients mean oxysterol levels (±SD) (from a median of 2.5 samples per individual) were above suggested cut-off levels (24.5 for cholestane-3β,5α,6β-triol (3β,5α,6β-triol); 47.5 for 7-ketocholesterol (7-KC)) [range: 3β,5α,6β-triol: 29-152; 7-KC: 78-390]. For 10 of the 16 individuals a total of 19 blood samples had been obtained at dates for which also neuro-psychiatric status was scored. For these 10 individuals means for both, plasma oxysterols and NPC-cdb scores were generated from up to three consecutive measurements per patient, and correlation analysis was performed by determining linear regression curves (Additional file 4: Figure S1).

## Results

### Generation of the NP-C clinical database (NPC-cdb)

With the aim to establish a comprehensive repository (NPC-cdb) to store, share and analyze diagnostic and clinical findings in NP-C patients, we queried the literature for symptoms of direct and possible disease-relevance. To systematically assess whether and when in the course of disease these symptoms manifest, we designed a questionnaire of 270 questions covering 12 subject areas, including diagnostics, pre/postnatal development, visceral, neurological and psychiatric signs, speech and auditory function, social skills and education, therapy and family history (Additional file 1). This questionnaire assisted to acquire longitudinal data in personal interviews that were entered as fixed terms and/or quantifiable parameters into a customizable electronic database (Additional file 2).

### Diagnostic findings in 42 patients support the heterogeneous nature of NP-C

**Figure 2:**
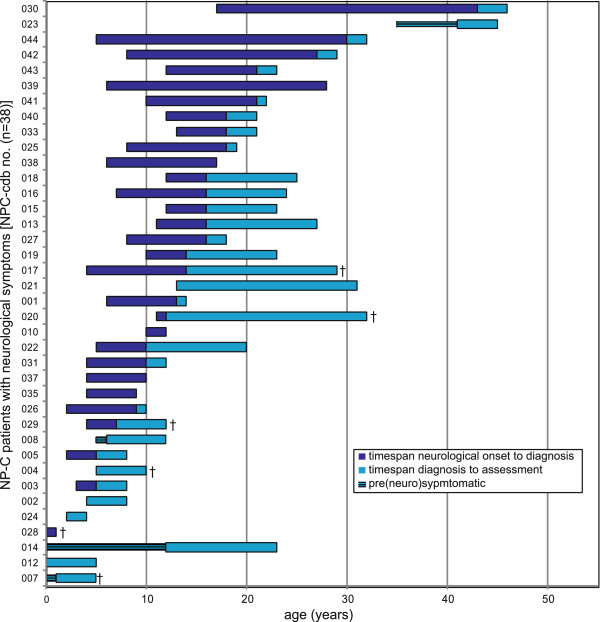
**Spectrum of disease presentation in studied neurological NP-C patient cohort (n=38).** Horizontal bars show disease duration (in years) in individual patients (represented by NPC-cdb no.; see Figure 1) from the onset of neurological symptoms until the age at data assessment. Dark blue, timespan from first reported neurological sign until diagnosis NP-C. Light blue, timespan from diagnosis until data assessment. In four patients NP-C was diagnosed prior to the onset of neurological signs (narrow bars). Deceased patients are highlighted by crosses. Patients are aligned according to age at diagnosis.

To test the applicability of NPC-cdb, we obtained diagnostic and longitudinal clinical findings from the majority of NP-C families in Germany and Switzerland. Comprehensive datasets from 42 patients were analyzed, among them six sib-pairs and one sib-trio (Figure 1). The age of the patients at time of assessment ranged from newborn to 46 years (mean=17.4±11.2 y) (Figure 2). Eight patients died since assessment, of them two with hepatic failure and six in a progressed neurodegenerative state. 34 patients received Miglustat (Zavesca^©^, Actelion Pharmaceuticals) for treating neurological manifestations [4], however few were on treatment for more than one year (mean=1.8±1.6 y). Of the eight untreated patients, five were in a progressed disease stage, two freshly diagnosed and one did not show neurological signs. While only a minority of patients (n=9) reported to take dietary supplements, most received symptomatic treatment such as physical (n=25) or speech therapy (n=22).

The mean delay from first neurological symptom to diagnosis NP-C was 5.7±7.6 years (Figure 1, Figure 2), supporting data from other cohorts that diagnosing NP-C is difficult. Of the 26 patients in whom filipin staining of cultured fibroblasts was performed to support or exclude NP-C as diagnosis, 17 (65%) showed a “classical” cholesterol storage pattern compatible with NP-C. In eight patients (31%) filipin staining pattern was “variant” [3,19,20], and in patients #027 and #029, albeit diagnosed at a progressed disease stage, even reported as indifferent to healthy controls. In contrast, all 16 patients (100%) in which plasma oxysterols had been determined showed elevated cholestane-3β,5α,6β-triol and 7-ketocholesterol levels compatible with NP-C.

Molecular analyses of *NPC1* and *NPC2* genes identified likely disease-causing homozygous or compound-heterozygous DNA sequence variants in 36 (86%) patients, among them one NPC2-patient. In six patients (14%) with typical clinical and/or biochemical signs of NP-C, sequencing identified either a single (n=5) or no (n=1; #010) mutations at the known disease loci. Of the in total 80 mutant alleles, 62 (78%) carried missense variants, two affected splicing, five introduced a pre-term stop-codon, nine were deletions and two insertions (Figure 1).

Based on the clinical data acquired, we retrospectively assessed the likelihood that a patient would have been considered as NP-C when a recently introduced clinical Suspicion Index (SI) score [8] would have been applied at the time of diagnosis. According to this score, testing for NP-C would have been recommended for only 30 (70%) of our patients (mean±SD SI score: 173±50), and critical follow-up for 7 (17%; mean±SD SI score: 52±24), respectively. Remarkably, based on the SI score NP-C would have been considered as unlikely in five patients (#002,007,014,023,028) (13%; mean±SD SI score: 16±9) (Figure 1). Importantly, four of these five patients presented with isolated splenomegaly or hepatosplenomegaly, while NP-C typical neurological signs in this subcohort manifested if at all only shortly before diagnosis or later (mean interval from onset of neurological signs to diagnosis=0.8±1.8 y). This suggested low sensitivity of the SI score in patients with predominatly visceral disease or early neuropsychiatric disease stages. We tested this hypothesis by comparing patients with a long delay between first neurological symptom and diagnosis to patients with a short disease history (first neurological symptom <3 y before diagnosis). Indeed, sensitivity of the SI score correlated negatively with duration of disease (p<3.9x10^-4^; Fisher’s exact test). These findings further accentuate the need for better strategies to identify patients early in the course of disease, such as 18 patients of our cohort (43%) that presented with prolonged jaundice and 9 (21%) with isolated organomegaly as neonates, long before typical neurological NP-C signs manifested.

### Cognitive and coordination deficits are early indicators of NP-C

**Figure 3:**
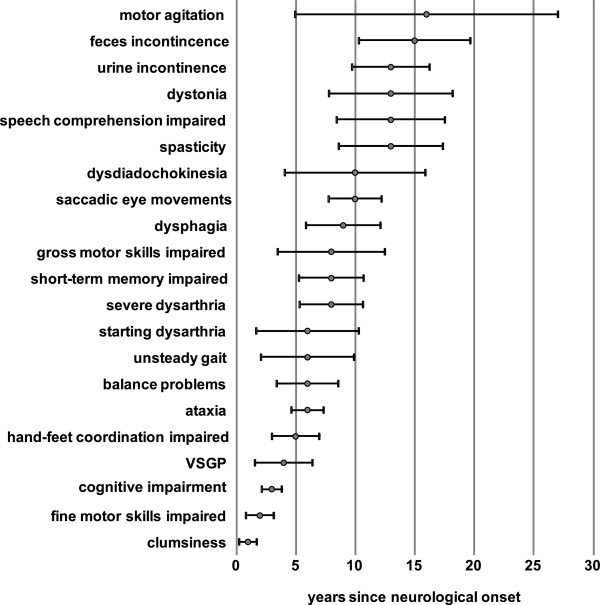
**Sequence of most frequent neuropsychiatric symptoms during disease course.** Estimated median time until presentation (±95%CI) of the 21 most frequent neurological signs present in >40% of studied neurological patients (n=38), normalized to the relative age at neurological disease onset (x=0). Symptoms are aligned in order of appearance (bottom to top). *VSGP*, vertical supranuclear gaze palsy.

One possibility to use longitudinal data acquired through NPC-cdb is to investigate how neurological disease as the major disease burden develops in NP-C patients. The majority of our patients (n=40) developed initial specific or less specific neuropsychiatric signs before adulthood (range: 0-41 y; mean=7.9±6.9 y) (Figure 2, Figure 1). At data assessment, only four patients (two neonates and two siblings of more severely affected sisters) did not show neuropsychiatric signs, making neurological involvement in NP-C equally prevalent as past or present splenomegaly (90%; n=38). However, while in most patients the exact onset and evolvement of organomegaly was difficult to assess, interview data joint with medical records allowed us to rather precisely reconstruct neurological disease. Of the 72 neuropsychiatric signs analyzed and consistent with previous studies [1,4], cognitive impairment (86%), coordination deficits (clumsiness 81%, ataxia 79%, impaired fine motor skills 79%), dysarthria (81%) and vertical supranuclear gaze palsy (VSGP, 76%) were reported as the most prevalent neuropsychiatric symptoms (Additional file 5: Table S1). Using censored Kaplan-Meier curves we modeled the manifestation of all 72 neuropsychiatric symptoms over the course of disease. Importantly, cognitive impairment (3.4 y, 95%CI 2.5-4.3), clumsiness (3.7 y, 95%CI 1.6-5.9) and impaired fine motor skills (3.4 y, 95%CI 1.6-5.9) were predicted as the earliest signs of neurological manifestation in our cohort (Figure 3). With this, these less specific symptoms ranked considerably more early than VSGP (7.4 y, 95%CI 4.7-10.8), ataxia (7.5 y, 95%CI 4.7-7.4) or dysarthria (6.9 y, 95%CI 4.8-9.1), neurological signs that are more generally known as associated with NP-C [1-4,12].

**Figure 4:**
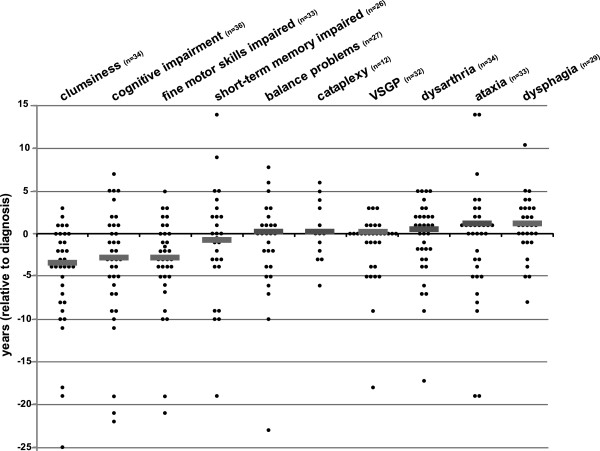
**Manifestation of 10 characteristic neurological NP-C signs relative to diagnosis.** Sequence of occurrence of 10 selected neurological symptoms with diagnostic importance (acc. to Wijburg et al., 2012, [8]), normalized to the relative age at diagnosis (y=0). Each dot represents one patient. Medians are indicated by grey bars. Symptoms are aligned in order of appearance (bottom to top). *VSGP*, vertical supranuclear gaze palsy.

As the early diagnosis and start of treatment is believed to benefit treatment outcome in NP-C [8-10], we determined the manifestation of selected neuropsychiatric signs relative to the time point of diagnosis (Figure 4). Notably, once symptoms considered as typical for NP-C were observed in a patient, the delay until diagnosis was short (mean delay from manifestation to diagnosis: VSGP -1.4±0.7 y, cataplexy 0.3±1.0 y, dysarthria 0.0±0.7 y). This indicated that the presence of such “classical” NP-C symptoms either succesfully initiates the right path to diagnosis, or that these signs are recognized immediately following the diagnosis NP-C in a patient. By contrast, delay from onset to diagnosis was long for less specific neuropsychiatric signs affecting cognition and coordination that in the vast majority of patients (>79%; Additional file 5: Table S1) was noticed considerably more early during disease course (e.g., clumsiness (-4.9±1.1 y), cognitive impairment (-3.6±1.1 y), impaired fine motor skills (-2.2±0.9 y), balance problems (-1.4±1.1 y) (Figure 4). These findings strongly propose that less specific neuropsychiatric signs are frequent and early indicators of NP-C, and that raising the awareness of this may help to substantially reduce the timespan until a patient is diagnosed.

### NP-C progression is variable and likely correlates with external and/or genetic factors

**Figure 5:**
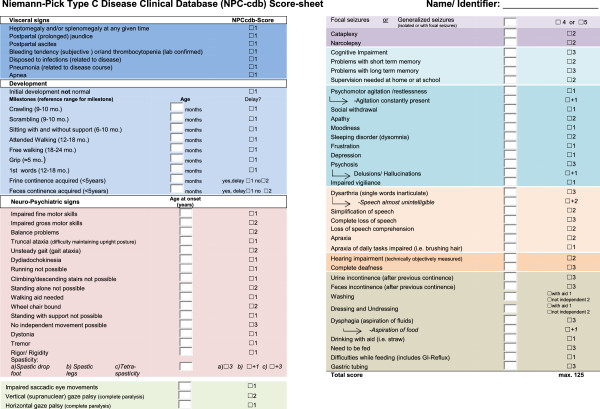
**Symptoms considered for NP-C clinical database (NPC-cdb) score.** Colored areas represent different subject categories in NPC-cdb questionnaire and database (Additional file 1 and Additional file 2).

NP-C manifestation and course appeared to vary considerably within our cohort (Figure 1, Figure 2). Several scoring systems have been developed to monitor disease progression in NP-C [10,13,17,23]. However, none of the existing tools covered the comprehensive patient-specific clinical information available through NPC-cdb. Moreover, previous scales quantify deterioration or improvement within defined symptom domains, which necessitates complete evaluation of all such domains at each visit. To ease the acquisition and documentation of comprehensive longitudinal data, we therefore established a novel clinical outcome scale (NPC-cdb score). Unlike previous tools, the NPC-cdb score represents the sum of all past and current symptoms present in a patient at a given time point, with each symptom (as listed in Figure 5) contributing a severity-weighted summand (for guidelines on how to apply the NPC-cdb score, see Additional file 3). For 10 patients in which plasma oxysterol levels were measured at visits for which scoring could be performed, scores correlated well with mean 3β,5α,6β-triol and 7-KC levels, which supports the hypothesis that the NPC-cdb score is a reliable indicator of NP-C severity (Additional file 4: Figure S1).

**Figure 6:**
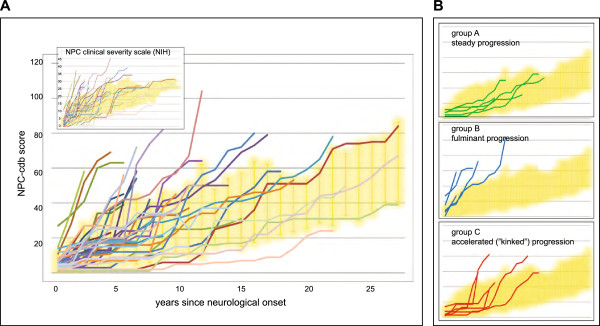
**Disease progression in 38 NP-C patients with neurological disease. (A)** Occurrence of disease symptoms over time (in years) was scored for each of the 38 patients (colored lines) individually with both, the NPC-cdb score (large graph) and the NIH clinical severity scale (Yanyanin et al., 2010, [17]; inset), each normalized to the age at neurological disease onset (x=0). Higher score values (y-axis) reflect higher number of symptoms. Standard deviations around mean values (reflecting the normal range) are represented by yellow clouds. **(B)** Examples for patients with particular types of disease progression. *Top*, steady disease course; *center*, rapid degenerative course; *bottom*, initial steady progression is followed by acceleration.

We applied the NPC-cdb score to retrospectively assess disease history in each of the 38 patients with neuropsychiatric signs. Consistent with previous findings [17], NP-C severity progressed in a close to linear fashion when the overall cohort was considered (Figure 6A; mean linear regression curve: y=1.83x+8.9; R^2^=0.96). Interestingly, however, and irrespective of whether the NPC-cdb score or a previously established clinical severity scale [17] were applied (Figure 6A, inset), disease progression independent of age of onset varied considerably: While most patients (n=24) progressed gradually over time (Figure 6B, top), seven patients with early neurological onset showed a fulminant progression with all annual NPC-cdb scores above the respective means of the collective (Figure 6B, center). Moreover, a further cluster of six patients with initially rather stable disease revealed a close to three-fold (2.9±1.1) accelerated progression at a later time point during disease (mean delay=6.7±2.5 y after neurological onset; Figure 6B, bottom). We questioned what might be possible reasons for such accelerated deterioration. Based on NPC-cdb, in four of these six patients seizures had occurred for the first time in the year preceding the rapid decline, while the prevalence of seizures in the overall cohort was 35% (n=15). Indeed, association analysis showed that despite correction for seizure-associated score points, patients showed a high likelihood for an accelerated disease progression within two years upon the first seizure event (p<7x10^-4^; Fisher’s exact test). This suggests that the occurrence of seizures is predictive for a poor prognosis in a patient.

We further questioned whether some of the phenotypic differences could be due to genetic reasons. However, due to the still small sample size and large number of *NPC1/NPC2* alleles in our cohort, no significant correlations between genotype at the known NP-C loci and overall disease progression were reached. However, consistent with previous observations [3] and irrespective of the age of onset, progression of disease was similar between NP-C siblings who shared the same haplotypes (Figure 1, Additional file 6: Figure S2), indicating that at least part of the variability in NP-C progression is associated with distinct *NPC1-* and *NPC2*-mutations.

## Discussion

We have developed the NP-C clinical database (NPC-cdb) with the aim to provide a unifying framework for clinical and diagnostic datasets on NP-C from multiple patient cohorts. A detailed, customized questionnaire facilitates to comprehensively obtain current and past medical history of NP-C patients that can be entered as fixed terms and/or quantifiable parameters into a decentralized electronic database. NPC-cdb is intuitive, can be customized to an individual study’s needs and allows for an easy sharing of distinct, anonymized datasets among researchers and clinicians from diverse disciplines. We envision NPC-cdb to become a starting point for large-scale collaborative studies on NP-C pathogenesis and on the efficiency of current and future therapies.

As a proof-of-concept, we applied our infrastructure to retrospectively assess disease progression in 42 NP-C patients of German and Swiss origin, to date the most comprehensive survey on NP-C in these countries. We confirm many of the findings in other cohorts [8,9,12-18], among them a delay of several years from neurological onset to diagnosis; difficulties to unambiguously secure NP-C with current diagnostic tools; or a striking influence of the age at onset on disease progression. However, systematic acquisition of the temporal sequence of clinical signs as assisted by NPC-cdb opens perspectives beyond this previous knowledge. For instance, while the frequently insidious onset of NP-C is known among experts [4,17], our data show for the first time in a larger cohort that unspecific neuropsychiatric signs involving cognitive, behavioral and motor skills are among the first disease indicators. Specifically, subtle signs such as clumsiness or perturbed fine motor skills were noticed in our cohort close to four years before distinct NP-C indicators such as VSGP, cataplexy or ataxia were reported. Certainly, our data do not exclude the possibility that also specific NP-C signs arise early in the course of disease, but are difficult to assess (such as impaired saccadic eye movements; [4]) and therefore may simply have been overlooked. Moreover, a substantial fraction of our data was acquired in personal interviews with non-expert relatives. As the earliest abnormalities reported by close family members are by nature subjective and often not documented as such in medical records, we can neither exclude a bias towards more familiar symptoms nor an interviewer-bias by retrospectively asking for the presence of a respective symptom. Nevertheless, our finding that less specific neuropsychiatric signs are early indicators of NP-C is supported by (i.) the large number of patients from all age groups with a similar sequence of clinical events and (ii.) a high congruency between interview and chart data for better documented NP-C symptoms (not shown). Importantly, our data also show that the occurrence of certain more specific NP-C signs appeared to pave the way for a diagnosis within less than 1.4 years after onset of the respective symptom. Our study therefore strongly suggests that to accelerate diagnosis, less specific neuropsychiatric signs should receive a higher attention.

This is of particular importance since even latest diagnostic aids to decide which patients should undergo NP-C testing strongly rely on the presence of the most prominent NP-C signs. For instance, a recently published retrospective chart review in a large cohort [8] revealed that cholestasis, splenomegaly, VSGP, cataplexy, cognitive decline, co-affected relatives and a co-occurrence of symptoms from different disease domains are the strongest NP-C predictors. Consequently, a proposed Suspicion Index (SI) tool weighs these symptoms relatively high, while frequent, but less specific motor coordination, cognitive and behavioral problems are ranked as moderate to low indicators. While also our study shows that the SI tool is very sensitive in identifying patients that have readily apparent disease, it has not been taken into account during the generation how far in the disease process in individual patients had already progressed before diagnostic testing was initiated. Retrospective application of this Suspicion Index tool to our collective revealed that at diagnosis 30% of our patients would not have been considered as among the strongest candidates for NP-C testing, while NP-C would have been regarded even as unlikely in 13%. Importantly, diagnosis in this sub-cohort was secured before or early after onset of first neurological symptoms, which is reflected by a negative correlation between Suspicion Index score and time to diagnosis. With this, our data take into question that the Suspicion Index tool will significantly contribute to identify NP-C patients early in the disease process. Instead, to reduce diagnostic delay and with this raise response to treatment [8-10], we propose that less specific NP-C indicators should be included in a decision to initiate NP-C testing. This is of particular relevance as plasma oxysterols as a rapid and reliable biochemical screening tool may soon enable the cost-efficient identification of putative NP-C patients from larger cohorts with diverse clinical backgrounds.

For studying the temporal development of NP-C we generated the NPC-cdb score, which allowed us to closely monitor disease progression independent from the age of onset. Unlike a similarly comprehensive clinical severity scale [17], determining the NPC-cdb score for a patient does neither rely on laboratory investigations nor on laborious re-evaluation of distinct clinical sub-domains at each visit. Instead, the sum of all severity-weighted clinical symptoms present at distinct time points during the course of disease is determined once from questionnaire data obtained at one comprehensive data assessment. During follow-up visits, only symptoms that manifested since the previous data entry are to be added. This ease of use should proof useful in clinical settings and with this complement widely used, but less comprehensive scales that only poorly reflect the heterogeneous clinical picture of NP-C [10,12,13,23]. As the NPC-cdb score is in principle open ended, further symptoms of interest can easily be added which may be of particular interest for prospective and interventional studies. Our focus here was on a solid reconstruction of disease history. Interestingly and in seeming conflict with another cohort where NP-C severity progressed in a close to linear fashion [17], a sizable fraction of our patients (n=6; 14%), among them one sib pair, showed a steep deterioration of abilities after an initially only slowly-progressive disease. In our cohort this deterioration was associated with seizures, onset of which in a patient could signalize progressed neurological state. Alternatively, seizures might as well impair NP-C relevant brain functions, which secondarily accelerates disease progression as reflected by higher NPC-cdb scores. Closer monitoring of clinical events before and after the onset of seizures in future studies may help to distinguish between cause and consequence.

In addition to epilepsy, the existence of further factors impacting on adverse or beneficial clinical outcomes is highly probable, but failed to be demonstrated here due to insufficient patient numbers. Experience from complex diseases propose that much larger, equally well-documented cohorts will be needed to unambiguously identify also factors with more subtle impact on disease expression [24]. This is particularly the case when aiming at the identification of genetic factors or biomarkers as the basis for particular NP-C phenotypes that may explain the heterogeneous nature of the disease. The prospect of Miglustat as a specific treatment option for NP-C [25] has triggered numerous trials in several national NP-C patient cohorts (e.g., [9,10,13-17,23,26]). These studies have contributed significantly to our understanding of which symptoms within the NP-C spectrum are frequent and specific. The systematic collection of comprehensive diagnostic and longitudinal clinical information in a central database will further extend and improve the generalizability of findings from individual cohorts. By providing large-enough patient numbers this may allow to precisely characterize the heterogeneous clinical picture and unambiguously test whether existing or future treatment strategies are efficient. We are confident that NPC-cdb may now provide a platform for such initiatives.

## Competing interest

MS, YA, SK, DM, EM and HR have received travel support and honoraria for presenting at events sponsored by Actelion Pharmaceuticals. EM and HR have received unrestricted research grants from Actelion Pharmaceuticals Deutschland GmbH. EM has received honoraria from serving on the scientific advisory board of Actelion Pharmaceuticals.

## Authors’ contributions

HR conceptualized and supervised the study; MS, ST, YA, HJ, SK, DO, EM and HR acquired, analyzed and/or interpreted data; MS, CF and HR performed statistical analyses; MS and HR wrote the manuscript. All authors read and approved the final manuscript.

## Supplementary Material

Additional file 1 (e-Questionnaire): NPC-cdb questionnaire. Click here for file

Additional file 2 (e-Database): NPC-cdb electronic version (based on Microsoft Office Access, vs. 2010). Click here for file

Additional file 3 (e-Guidelines): NPC-cdb score guidelines. Click here for file

Additional file 4: Figure S1 Correlation of NPC-cdb score with plasma oxysterol levels and filipin phenotype. For 10 NP-C patients from our cohort, mean plasma levels (±SD) of cholestane-3β,5α,6β-triol (3β,5α,6β-triol) and 7-ketocholesterol (7-KC) were determined according to Jiang et al. [22] from a total of 19 samples (with a median of 2.5 samples per individual) (y-axis). Mean NPC-cdb scores per patient were generated from phenotypic information acquired at up to three consecutive visits (x-axis). Higher score values (y-axis) reflect higher number of symptoms. Where available, “classic” or “variant” filipin staining pattern are highlighted in grey. Click here for file

Additional file 5: Table S1 Temporal development of 72 longitudinally-assessed NP-C symptoms (sorted acc. to frequency of occurrence). Click here for file

Additional file 6: Figure S2 Disease progression in siblings. Occurrence of disease symptoms over time (in years) was scored for each sibling of 3 NP-C sib pairs and one NP-C sib trio with the NPC-cdb score and normalized to the age at neurological disease onset (x=0). Higher score values (y-axis) reflect higher number of symptoms. Standard deviations (reflecting the normal range) are represented by shaded clouds. Click here for file
