# Supplementary material for: Niemann-Pick disease type C clinical database: cognitive and coordination deficits are early disease indicators
Source: Orphanet J Rare Dis. 2013 Feb 22;8:35. doi: 10.1186/1750-1172-8-35 (PMC3649939; doi:10.1186/1750-1172-8-35)
Supplement: Additional file 1 — (e-Questionnaire): NPC-cdb questionnaire. [file 1750-1172-8-35-S1.doc]

**NPC-cdb questionnaire**

Index patient

| Last name | First name | Date of birth | Place of birth | Sex | Identifier:  (e.g. NPCcdb-HD-001) |
| --- | --- | --- | --- | --- | --- |
|  |  |  |  | mf |  |

|  | Last name | First name | Date of birth | Place of birth | Sex | affected? |
| --- | --- | --- | --- | --- | --- | --- |
| mother |  |  |  |  |  |  |
| father |  |  |  |  |  |  |
| sibling #1 |  |  |  |  |  |  |
| sibling #2 |  |  |  |  |  |  |
| sibling #3 |  |  |  |  |  |  |
| sibling #4 |  |  |  |  |  |  |

| Profession/ highest level of education mother |  |
| --- | --- |
| Profession/ highest level of education father |  |

| **Contact details:** | | |
| --- | --- | --- |
| Street and No. | | |
|  | | |
| City | ZIP code | State/ Country |
|  |  |  |
| Phone private |  | |
| Phone mobile |  | |
| Fax |  | |
| Email |  | |

**Contact details: Attending physicians:**

| Name | | First name | Specialized in : |
| --- | --- | --- | --- |
|  | |  |  |
| **Address:** | | | |
| Street and No. | | City | ZIP code |
|  | |  |  |
| **Phone:** | | | |
| Private | Mobile | Fax | Email |
|  |  |  |  |

| Name | | First name | Specialized in/ NPC Center: |
| --- | --- | --- | --- |
|  | |  |  |
| **Address:** | | | |
| Street and No. | | City | ZIP code |
|  | |  |  |
| **Phone:** | | | |
| Private | Mobile | Fax | Email |
|  |  |  |  |

1. **Diagnosis**

| 1. Diagnosis NP-C confirmed at (date) |  |
| --- | --- |
| 1. Age at diagnosis | years |

| 1. Course of diagnostic evaluation  (first symptoms **→**diagnosis NPC) |  |
| --- | --- |
| 1. Presenting symptom(s) |  |
| 1. Additional diseases present/ clinical findings |  |
| 1. Differential diagnosis considered during diagnostic evaluation |  |

| 1. First neurological signs | 1. First visceral signs |
| --- | --- |
| perinatal at birth | perinatal at birth |
| early infantile <2 years | early infantile <2 years |
| late infantile 3-5 years | late infantile 3-5 years |
| juvenile 5-16 years | juvenile 5-16 years |
| adult >16 years | adult >16 years |

**Laboratory Diagnostics**

**Genetic analyses/DNA** testing

| 1. DNA testing performed? | Yes | No | Unknown |
| --- | --- | --- | --- |
| 1. NPC1 mutations identified | Yes | No | Unknown |
| 1. Mutation #1 NPC1 /mutation #2 NPC1 |  | |  |
| 1. NPC 2 mutations identified | Yes | No | Unknown |
| 1. Mutation #1 NPC2 /mutation #2 NPC2 |  | |  |
| 1. DNA testing performed at/by (place) |  | | |
| 1. DNA testing performed on (date) |  | | |
| 1. Further genetic variants /SNPs identified |  | | |
| 1. DNA available/stored | Yes | No | Unknown |
| 1. DNA available in/ from |  | | |

**Fibroblast** testing

| 1. Filipin-test performed? | Yes | | No | | Unknown |
| --- | --- | --- | --- | --- | --- |
| 1. Results of Filipin-test | Classic | | Variant | | Normal |
| 1. Place of Filipin-test |  | | | | |
| 1. Date of Filipin-test |  | | | | |
| 1. Cholesterol esterification determined | Yes | | No | | Unknown |
| 1. Results of cholesterol esterification (%ctrl) |  | | | | |
| 1. Place of cholesterol esterification assay |  | | | | |
| 1. Date of cholesterol esterification assay |  | | | | |
| 1. Fibroblasts available/stored | Yes | No | | Unknown | |
| 1. Fibroblasts available in/ from |  | | | | |

**Blood tests**

| 1. Enzymatic activities analyzed (e.g. Chitotriosidase) | Yes | No | | Unknown |
| --- | --- | --- | --- | --- |
| 1. Results of enzymatic assays (nmol/ml/h) |  | | | |
| 1. Place of enzymatic assays |  | | | |
| 1. Date of enzymatic assays |  | | | |
| 1. Oxysterols measured | Yes | No | | Unknown |
| 1. Results of Oxysterols (7KC/ Ch 3,5,6) |  | |  | |
| 1. Place Oxysterol measurement |  | | | |
| 1. Date of oxysterol measurement |  | | | |
| 1. Blood sample/serum stored: | Yes | No | | Unknown |
| 1. Blood sample/serum available in/from: |  | | | |

Further tests

| 1. CT-scan(s) | Yes, obtained on: | No | Results: |
| --- | --- | --- | --- |
| 1. MRI-scan(s) | Yes, obtained on: | No | Results: |
| 1. NMR-scan(s) | Yes, obtained on: | No | Results: |
| 1. Trail making[[1]](#footnote-2) | Yes, obtained on: | No | Results:  Easy (e.g. connect A-B-C):  Difficult (e.g. connect A-1-B-2-C-3): |
| 1. Grooved Peg-Board test[[2]](#footnote-3) | Yes, obtained on: | No | Results: Dominant hand (right/left):  Not dominant hand (right/left): |
| 1. Further diagnostics / analyses / tests: | | | |

| 1. **Remarks on section “Diagnostics”** |
| --- |

1. **History of Pregnancy (Mother)**

| 1. Number of pregnancies |  | | |
| --- | --- | --- | --- |
| 1. Number of children |  | | |
| 1. Number of miscarriages |  | | |
| 1. Multiple pregnancies | Yes | No | *Numbe*r |

| 1. Cause of miscarriage(s) | Induced | Not induced |
| --- | --- | --- |
| 1. Week of pregnancy of miscarriage |  | WOP |

**Medication during pregnancy**

| 1. Medication/ Drug | *Drug1:* | *Drug2:* | *Drug3:* |
| --- | --- | --- | --- |
| 1. Frequency of intake | /day | /day | /day |
| 1. Duration of intake |  |  |  |
| 1. Dosage |  |  |  |

| 1. Maternal smoking | Yes | No |
| --- | --- | --- |
| 1. Alcohol intake | Yes | No |
| 1. Drug intake | Yes | No |

**Ultrasound examinations**

| 1. 1^st^ trimester amniotic fluid | | | normal | | vol. reduced | vol. increased | | not done/ unknown | |
| --- | --- | --- | --- | --- | --- | --- | --- | --- | --- |
| 1. 2^nd^ trimester amniotic fluid | | | normal | | vol. reduced | vol. increased | | not done/ unknown | |
| 1. 3^rd^ trimester amniotic fluid | | | normal | | vol. reduced | vol. increased | | not done/ unknown | |
|  |  |  | |  | | |  | |  |
| 1. 1^st^ trimester CRL[[3]](#footnote-4)/ length | | | normal | | too small | too large | | not done/ unknown | |
| 1. 2^nd^ trimester CRL/ length | | | normal | | too small | too large | | not done/ unknown | |
| 1. 3^rd^ trimester CRL/ length | | | normal | | too small | too large | | not done/ unknown | |

| 1. 1^st^ trimester organ abnormalities | Yes | No | not done/ unknown |
| --- | --- | --- | --- |
| 1. 2^nd^ trimester organ abnormalities | Yes | No | not done/ unknown |
| 1. 3^rd^ trimester organ abnormalities | Yes | No | not done/ unknown |

| 1. 1^st^ trimester remarks |  |
| --- | --- |
| 1. 2^nd^ trimester remarks |  |
| 1. 3^rd^ trimester remarks |  |

| 1. Prenatal testing performed? (on NP-C) | Yes | No |
| --- | --- | --- |
| 1. Maternal serum screening | Yes | No |
| 1. Chorionic villus sampling | Yes | No |
| 1. Amniocentesis | Yes | No |
| 1. Further prenatal analyses performed? |  | |

Birth

| 1. Vaginal (spontaneous) delivery   **NPC-cdb-score** | 1. Caesarean Section |
| --- | --- |

| 1. Complications during birth? | Yes | No | |
| --- | --- | --- | --- |
|  |  | | |
| 1. Length of pregnancy | WOP | | |
| 1. Preterm labor | Yes | | No |
| 1. Amniotic fluid aspiration | Yes | | No |

| 1. Weight at birth |  | | |
| --- | --- | --- | --- |
| 1. Length at birth |  | | |
| 1. Head circumference |  | | |
| 1. APGAR[[4]](#footnote-5) | 1^st^ min | 5^th^ min | 10^th^ min |
|  |  |  |

**Primary examination of the infant**

|  |  |  |  | |
| --- | --- | --- | --- | --- |
| 1. Postpartal jaundice | Yes | No |  | 1 |
| 1. Postpartal ascites | Yes | No |  | 1 |
| 1. Bilirubin level (μmol/l) |  | Not tested |  | - |
| 1. Bili lights | Yes | No |  | - |
| 1. Postpartal lung failure | Yes | No |  | - |

| 1. **Remarks on the section “Pregnancy**”: |
| --- |

1. **Visceral Symptoms**

| **NPC-cdb-score** |  | | | size (unit) |  | |
| --- | --- | --- | --- | --- | --- | --- |
| 1. Hepatomegaly present at diagnosis | | Yes | No |  |  | 1^*^ |
| 1. Hepatomegaly present at current visit | | Yes | No |  |  |
| 1. Splenomegaly present at diagnosis | | Yes | No |  |  |
| 1. Splenomegaly present at current visit | | Yes | No |  |  |

^*^Point should only be scored once, independent of the time and either for hepatosplenomegaly and/or splenomegaly

| 1. Bleeding tendency[[5]](#footnote-6)(subjective) | Yes | No |  | 1^#^ |
| --- | --- | --- | --- | --- |
| 1. Thrombocytopenia[[6]](#footnote-7) (lab confirmed) | Yes | No |  |

***^#^*** Point should only be scored once

|  |  |  |  | |
| --- | --- | --- | --- | --- |
| 1. Disposed to infections | Yes | No |  | 1 |
| 1. Frequency of infections | /month | |  | - |
| 1. Infections of the upper airways | Yes | No |  |  |
| 1. Infections of the lower airways | Yes | No |  |  |
| 1. Enterogastritis | Yes | No |  |  |
| 1. Further infections |  | |  |  |
| 1. Pulmonary disease | Present at birth | |  | - |
|  | Yes | No |  |  |
|  | In course of disease | |  |  |
|  | Yes | No |  |  |
| 1. Pneumonia | Yes, age of onset  years | No |  | 1 |
| 1. Asthma | Yes, age of onset  years | No |  | - |
| 1. Apnea | Yes, age of onset  years | No |  | 1 |
| 1. Respiratory therapy interventions (O2, nebulizer, suction, PT[[7]](#footnote-8) vest, chest PT) | Yes, age of onset  years | No |  | - |
| 1. Abnormalities in chest X-ray | Yes | No |  | - |

| 1. Further visceral symptoms: |
| --- |

| 1. **Remarks on section “visceral symptoms”** |
| --- |

1. **Development**

| **NPC-cdb-score** |  |  |  | |
| --- | --- | --- | --- | --- |
| 1. Initial psychomotor development normal? | Yes | No |  | no=1 |
| 1. Age at first abnormalities | years | |  | |

| Milestone (reference range for milestone) | Age |  | ***each  delayed milestone*** |
| --- | --- | --- | --- |
| 1. Crawling (9-10 mo.) | months |  | 1 |
| 1. Scrambling (9-10 mo.) | months |  | 1 |
| 1. Sitting with and without support (6-10 mo.) | months |  | 1 |
| 1. Attended Walking (12-18 mo.) | months |  | 1 |
| 1. Free walking (18-24 mo.) | months |  | 1 |
| 1. Grip (~5 mo.) | months |  | 1 |
| 1. 1st words (12-18 mo.) | months |  | 1 |
| 1. 1st sentences (~24 mo) | months |  | 1 |
| 1. Urine continence acquired (<5 years) | months | not acquired | 1  2 |
| 1. Feces continence acquired (<5 years) | months | not acquired | 1  2 |

| 1. **Further remarks on development:** |
| --- |

1. **Neurology**

**NPC-cdb-score**

| 1. First neurological deficits (age) |  | |
| --- | --- | --- |
| 1. IQ tested | Yes | No |
| 1. IQ abnormal? | Yes | No |
| 1. IQ (e.g. acc. to WAIS) |  | |
| 1. Verbal fluency testing | No. words/min | |

|  |  |  |  | |
| --- | --- | --- | --- | --- |
| 1. Clumsiness/ impaired coordination | Yes,  age of onset | No |  |  |
| 1. Impaired fine motor skills | Yes,  age of onset | No |  | 1 |
| 1. Impaired gross motor skills | Yes,  age of onset | No |  | 2 |
| 1. Impaired coordination of hands and feet (i.e. when doing sports) | Yes,  age of onset | No |  |  |

| 1. Balance problems | Yes,  age of onset | No |  | 2 |
| --- | --- | --- | --- | --- |
| 1. Ataxia (incl. limb ataxia) | Yes,  age of onset | No |  |  |
| 1. Truncal ataxia | Yes,  age of onset | No |  | 1 |
| 1. Dysdiadochokinesia | Yes,  age of onset | No |  | 1 |

| 1. Unsteady gait | Yes,  age of onset | No |  | 2 |
| --- | --- | --- | --- | --- |
| 1. Running not possible | Yes,  age of onset | No |  | 1 |
| 1. Climbing stairs not possible | Yes,  age of onset | No |  | 1 |
| 1. Descending stairs not possible | Yes,  age of onset | No |  |
| 1. Walking aid | Yes,  age of onset | No |  | 1 |
| 1. Standing alone not possible | Yes,  age of onset | No |  | 2 |
| 1. Wheel chair bound | Yes,  age of onset | No |  | 2 |
| 1. Standing with help not possible | Yes,  age of onset | No |  | 1 |
| 1. No independent movement possible | Yes,  age of onset | No |  | 3 |

| 1. Dystonia | Yes,  age of onset | No |  | 1 |
| --- | --- | --- | --- | --- |
| 1. Tremor | Yes,  age of onset | No |  | 1 |
| 1. Rigor | Yes,  age of onset | No |  | 1 |

| 1. Spasticity | Yes,  age of onset | No |  |  |
| --- | --- | --- | --- | --- |
| 1. Spastic drop foot | Yes,  age of onset | No |  | *3[[8]](#footnote-9)* |
| 1. Spastic legs | Yes,   age of onset | No |  | *+1* |
| 1. Tetraspasticity | Yes,  age of onset | No |  | *+3* |

| 1. Muscular atrophy | Yes,  age of onset | No |  |  |
| --- | --- | --- | --- | --- |

| 1. Impaired saccadic eye movement | Yes,  age of onset | No |  | 1 |
| --- | --- | --- | --- | --- |
| 1. Vertical (supranuclear) gaze palsy | Yes,  age of onset | No |  | 2 |
| 1. Horizontal gaze palsy | Yes,   age of onset | No |  | 1 |

| 1. Seizures | Yes,  age of onset | No |  |  |
| --- | --- | --- | --- | --- |
| 1. Focal seizures | Yes,   age of onset | No |  | 4*^*^* |
| 1. Frequency (of focal seizures) | /Month | |  | |
| 1. Generalized seizures (Grand-mal) | Yes,  age of onset | No |  | *5^*^* |
| 1. Frequency (of generalized seizures) |  | /Month |  |
| 1. Duration of the last episode |  | Minutes |  |
| 1. Seizures only with trigger | Yes | No |  |
| 1. Febrile convulsions | Yes  age of onset | No |  |
| 1. Frequency (of febrile convulsions) | /month |  |  |
| 1. Absence seizures | Yes,  age of onset | No |  |

| 1. Cataplexy | Yes,  age of onset | No |  | 2 |
| --- | --- | --- | --- | --- |
| 1. Frequency of cataplexy | day/week/month | |  | |
| 1. Cataplexy only upon emotional excitement | Yes | No |  | |
|  |  |  |  | |
| 1. Narcolepsy | Yes,  age of onset | No |  | 2 |

| Medication for seizures | | | | Yes | No |
| --- | --- | --- | --- | --- | --- |
|  | Name of Medication | Dosage | Duration of intake (month/years) | Improvement under treatment | |
| 1. Drug 1 |  |  |  | Yes | No |
| 1. Drug 2 |  |  |  | Yes | No |
| 1. Drug 3 |  |  |  | Yes | No |

| 1. Last EEG | Abnormal | Normal | Not carried out |
| --- | --- | --- | --- |

| 1. **Remarks on section “Neurology”:** |
| --- |

1. **Cognitive abilities/ Behavior/ Psychiatric symptoms**

**NPC-cdb-score**

| 1. Loss of cognitive abilities | Yes,  age of onset | No |  | 3 |
| --- | --- | --- | --- | --- |
| 1. Problems with short time memory | Yes,  age of onset | No |  | 2 |
| 1. Problems with long term memory | Yes,  age of onset | No |  | 3 |
| 1. Supervision at home or at school needed[[9]](#footnote-10) | Yes,  age of onset | No |  | 2 |

| 1. Psychomotor agitation (in unfamiliar environment) | Yes,  age of onset | No |  | 1 |
| --- | --- | --- | --- | --- |
| 1. Agitation in familiar environment | Yes,  age of onset | No |  | +1 |
| 1. Social withdrawal | Yes,  age of onset | No |  | 1 |
| 1. Apathy | Yes,  age of onset | No |  | 2 |
| 1. Moodiness | Yes,  age of onset | No |  | 1 |
| 1. Sleeping disorder | Yes,  age of onset | No |  | 2 |
| 1. Initiating sleep disorder | Yes,  age of onset | No |  | - |
| 1. Maintaining sleep disorder | Yes,  age of onset | No |  | - |

| 1. Frustration | Yes,  age of onset | No |  | 1 |
| --- | --- | --- | --- | --- |
| 1. Depression | Yes,  age of onset | No |  | 1 |
| 1. Psychosis | Yes,  age of onset | No |  | 3 |
| 1. Delusions | Yes,  age of onset | No |  | *+1* |
| 1. Hallucinations | Yes,  age of onset | No |  |
| 1. Impaired vigiliance | Yes,  age of onset | No |  | 1 |

| 1. **Remarks on section “Behavior and cognitive abilities”:** |
| --- |

1. **Speech/ Hearing**

| **NPC-cdb-score** |  |  |  | |
| --- | --- | --- | --- | --- |
| 1. Dysarthria (Single words inarticulate) | Yes,  age of onset | No |  | 3 |
| 1. Whole sentences inarticulate | Yes,  age of onset | No |  | *+2* |
| 1. Loss of speech (beginning) | Yes,  age of onset | No |  | 2 |
| 1. Complete loss of speech | Yes,  age of onset | No |  | 3 |

| 1. Dysphasia (loss of speech comprehension) | Yes,  age of onset | No |  | 2 |
| --- | --- | --- | --- | --- |
| 1. Comprehension of simple requests impaired (i.e. take the ball) | Yes | No |  | - |
| 1. Comprehension of complex requests impaired (i.e. fetch your shoe and put it on) | Yes | No |  | - |

| 1. Apraxia/ Dyspraxia | Yes,  age of onset | No |  | 2 |
| --- | --- | --- | --- | --- |
| 1. Comprehension of daily tasks impaired (i.e. brushing hair) | Yes | No |  | +1 |
| 1. Comprehension of complex tasks impaired (i.e. tying shoes) | Yes | No |  | - |

| 1. Speech and language therapy (SLT) | Yes,  age of onset | No |  | - |
| --- | --- | --- | --- | --- |
| 1. Frequency of SLT |  | |  |

| 1. Hearing impairment (measured) | Yes,  age of onset | No |  | 2 |
| --- | --- | --- | --- | --- |
| 1. Deafness | Yes,  age of onset | No |  | 3 |
| 1. Hearing device | Yes,  age of onset | No |  | - |

| 1. **Remarks on section “Speech /Hearing”:** |
| --- |

1. **Daily routines**

|  |  |  |  | |
| --- | --- | --- | --- | --- |
| 1. Loss of urine continence | Yes,  age of onset | No |  | 3 |
| 1. Urge incontinence | Yes | No |  | - |
| 1. Overflow incontinence | Yes | No |  | - |
| 1. Loss of feces continence | Yes,  age of onset | No |  | 3 |

| 1. Defecation | alone | with aid | diapers |
| --- | --- | --- | --- |

|  |  | frequency | under therapy | |
| --- | --- | --- | --- | --- |
| 1. Diarrhea | Yes,  age of onset | /week | Yes | No |
| 1. Constipation | Yes,  age of onset | /week | Yes | No |
| 1. Meteorisms (gas bloat) | Yes,  age of onset | /week | Yes | No |

| 1. Washing | alone |  | - |
| --- | --- | --- | --- |
|  | with aid |  | 1 |
|  | not independent |  | 2 |
| 1. Dressing and Undressing | alone |  | - |
|  | with aid |  | 1 |
|  | not independent |  | 2 |

| 1. **Remarks on section “Daily routines”:** |
| --- |

1. **Eating and drinking**

|  |  | |  |  | |
| --- | --- | --- | --- | --- | --- |
| 1. Dysphagia | | Yes,  age of onset | No |  | 3 |
| 1. Aspiration of food | | Yes | No |  | *+1* |
| 1. Aspiration of drinks | | Yes | No |  | - |
| 1. Drinking | | alone | with aid or device (straw) |  | 1 (aid) |
| 1. Necessity of feeding | | Yes,  age of onset | No |  | 3 |
| 1. Difficulties while feeding | | Yes,  age of onset | No |  | 1 |
| 1. GI-reflux | | Yes,  age of onset | No |  | - |
| 1. Gastric tubing | | Yes,  age of onset | No |  | 3 |
| 1. Nutritional supplements | | Yes,  age of onset | No |  | - |

| 1. **Remarks on section “Eating and Drinking”:** |
| --- |

1. **Education and Social criteria**

|  | | | normal | integrative |
| --- | --- | --- | --- | --- |
| 1. kindergarden | Yes | No |  |  |
| 1. Elementary school | Yes | No |  |  |
| 1. School for mentally retarded children | Yes | No |  |  |
| 1. Special needs school | Yes | No |  |  |

| 1. **Level of education** | | | |
| --- | --- | --- | --- |
| Primary school | High school | college | no further school training |

| 1. **Job training** | | |
| --- | --- | --- |
| Yes | No | not old enough |
|  |  | attending not possible |

| 1. **Study at university** | | |
| --- | --- | --- |
| Yes | No | not old enough |
|  |  | attending not possible |

| 1. Profession |  |
| --- | --- |

| 1. Level of handicap (according to german care system) | % | age of determination | | |
| --- | --- | --- | --- | --- |
| 1. Level of care (according to german care system) | I  age | II   age | III   age | No level of care |

| 1. **Remarks on section “Education and Social criteria”:** |
| --- |

1. **Therapy**

| 1. Miglustat therapy (Zavesca®) | Yes,  begin with years | | No | |
| --- | --- | --- | --- | --- |
| 1. Dosage |  | | | |
| 1. Frequency of intake (per day) |  | | | |
| 1. Effect of Miglustat therapy | Improvement | Deterioration | | No change |
| 1. Observed beneficial effects of Miglustat |  | | | |
| 1. Negative effects of Miglustat |  | | | |

1. **Further medication**

| Name of medication | Start of therapy (age) | Reason for therapy | Dose mg | Frequency of intake (per day) | Improvement under therapy | | Side effects |
| --- | --- | --- | --- | --- | --- | --- | --- |
|  |  |  |  |  | Yes | No |  |
|  |  |  |  |  | Yes | No |  |
|  |  |  |  |  | Yes | No |  |
|  |  |  |  |  | Yes | No |  |
|  |  |  |  |  | Yes | No |  |
|  |  |  |  |  | Yes | No |  |
|  |  |  |  |  | Yes | No |  |
|  |  |  |  |  | Yes | No |  |
|  |  |  |  |  | Yes | No |  |

1. **Symptomatic therapy**

| 1. Physical therapy | Yes,   age at therapy start | No |
| --- | --- | --- |
| 1. Frequency of physical therapy |  |  |
| 1. Occupational therapy | Yes,   age at therapy start | No |
| 1. Frequency of occupational therapy |  | |
| 1. Lactose-free diet | Yes,    age at therapy start | No |
| 1. Disaccharide-free diet | Yes,   age at therapy start | No |
| 1. Loperamide | Yes,   age at therapy start | No |

| 1. Further therapy: |
| --- |

| 1. **Remarks on section “Therapy”:** |
| --- |

1. **Family history**

| 1. Parents consanguineous? | Yes | No |
| --- | --- | --- |
| 1. Ethnicity of family |  | |

| Diseases in family | Family member affected: |
| --- | --- |
| 1. GI/hepatic disease   e.g. hepatosplenomegaly, Crohn ´s disease |  |
| 1. Cardiovascular disease   e.g. myocardial infarction |  |
| 1. Metabolic disease   e.g. Diabetes mellitus |  |
| 1. Psychiatric disease   e.g. autism |  |
| 1. Neurological disease   e.g. Parkinson, Multiple Sclerosis, Alzheimer |  |
| 1. Cancer   e.g. familial breast cancer |  |
| 1. Autoimmune disease |  |
| 1. Mental retardation |  |

| 1. Further diseases in family | Family member affected: |
| --- | --- |
|  |  |
|  |  |
|  |  |

| 1. **Remarks on section “Family history”:** |
| --- |

| **Further Remarks /additional comments on patient:** |
| --- |

1. Tombaugh, T.N.T.N (2004). ["Trail Making test A and B: Normative Data Stratified by Age and Education"](http://search.proquest.com/docview/71715116?accountid=28041). *Archives of Clinical Neuropsychology : The Official Journal of the National Academy of Neuropsychologists* **19** (2): 203–214. [↑](#footnote-ref-2)
2. Rourke et al “Neuropsychological significance of lateralized deficits on the Grooved Pegboard test for older children with learning disabilities”. [*J Consult Clin Psychol.*](http://www.ncbi.nlm.nih.gov/pubmed/4726695)1973 Aug;41(1):128-34. [↑](#footnote-ref-3)
3. CRL crown-rump length [↑](#footnote-ref-4)
4. Abbreviation for **A**ppearance, **P**ulse, **G**rimace, **A**ctivitiy, **R**espiration after birth [↑](#footnote-ref-5)
5. Subjective judgments of the patient or family [↑](#footnote-ref-6)
6. May support or confound the estimation of laboratory results, may also present in patients without bleeding tendency [↑](#footnote-ref-7)
7. *PT*, physical therapy [↑](#footnote-ref-8)
8. *Focal seizures should be scored with 4 points, Generalized seizures (isolated or in combination with focal seizures) with 5 points, Absence seizures and febrile convulsions should be counted as generalized seizures [↑](#footnote-ref-9)
9. after previous independence [↑](#footnote-ref-10)
