# Supplementary material for: Niemann-Pick disease type C clinical database: cognitive and coordination deficits are early disease indicators
Source: Orphanet J Rare Dis. 2013 Feb 22;8:35. doi: 10.1186/1750-1172-8-35 (PMC3649939; doi:10.1186/1750-1172-8-35)
Supplement: Additional file 3 — (e-Guidelines): NPC-cdb score guidelines. [file 1750-1172-8-35-S3.pdf]

# NPC-cdb score Guidelines

## I. Background

This short manual aims to provide a guideline on how to evaluate clinical disease status in patients with Niemann-Pick disease type C (NP-C) using the **NPC-cdb score**. For details on the establishment of this clinical outcome measure, examples for its application and citation in future studies, please refer to the following publication:

Miriam Stampfer, Susanne Theiss, Yasmina Amraoui, Xuntian Jiang, Sigrid Keller, Daniel S. Ory, Eugen Mengel, Christine Fischer and Heiko Runz*
**Niemann-Pick disease type C clinical database: Cognitive and coordination deficits are early disease indicators.**
*Orphanet Journal of Rare Diseases* (2013).
*Contact: heiko.runz@med.uni-heidelberg.de

Note: Please note that the precise reconstruction of a phenotypically complex disorder such as NP-C by a numerical scoring system is challenging and must imply simplification. To establish the NPC-cdb score, the authors have thoroughly considered all clinical features present in their study cohort and included further symptoms from the literature. Suggestions on how to further improve accuracy and usability of this clinical outcome measure, as well as additional user feedback are strongly encouraged.

February 2013
Miriam Stampfer and Heiko Runz

## II. General remarks

The NPC-cdb score aims to reflect clinical status in a patient suspected or diagnosed with NP-C as comprehensively as possible. For this, it represents the sum of all current and past symptoms considered as disease-relevant that present(ed) in a patient by the time of assessment. Each symptom present (incl. any prior time point during disease course) contributes a severity-weighted summand (range: 1-5) to an overall outcome score ("NPC-cdb score"). Increase in score points is supposed to reflect reduction in abilities. For visits following the initial data assessment and scoring, only symptoms that manifested since the previous data entry are to be added (e.g. by using the *NPC-cdb Score-sheet*; Stampfer et al., 2013, Figure 4). This ease of use should proof helpful even in routine clinical settings. Scoring can be performed solemnly based on clinical and anamnestic information obtained at a patient's visit; no laboratory investigations need to be considered. To accommodate for future adjustments (e.g., the inclusion of additional symptoms considered as disease-related) the score is (in principle) open-ended (i.e., additional symptoms can be added at will). Unlike in previous scores (e.g., Itturiaga et al., 2006; Yanjanin et al., 2010), definition of clinical subdomains has been avoided; however, to ease assessment the NPC-cdb Score-sheet has been structured into the following

ten subject areas (as represented on the Score-sheet by different colors): 1.) visceral signs, 2.) development, 3.) motor functions, 4.) ocular-motor abnormalities, 5.) seizures/ cataplexy/ narcolepsy, 6.) cognitive abilities and memory, 7.) behavioral and psychiatric abnormalities, 8.) speech, 9.) hearing and 10.) abilities in daily life. To allow for longitudinal follow-up, the age at onset should be recorded for each symptom (on the NPC-cdb Score-sheet e.g. in the text field next to the symptom).

## III. Subject Areas

### 1.) Visceral signs

Seven visceral symptoms, each contributing one score point, are included in the NP-C score:

1. Hepatomegaly/ Splenomegaly/ Hepatosplenomegaly ☐1
   (above age-specific norms as evaluated by palpation or imaging at any time during history)
2. Postpartal prolonged jaundice ☐1
   (visible jaundice or laboratory-documented hyperbilirubinemia for typically >14 days after birth)
3. Postpartal/neonatal ascites ☐1
   (ascites as evaluated by palpation or imaging in perinatal/neonatal period)
4. Bleeding tendency/ thrombocytopenia ☐1
   (as evaluated by inspection or by report of increased bruising, nose bleeding,… or respective laboratory findings; no scoring if causes are unrelated to NP-C, e.g. due to alternative genetic condition/ hemophilia)
5. Disposed to infections ☐1
   (above age norm as reported or documented, incl. infections secondary to NP-C such as due to lack of mobility, aspiration, incontinence; no scoring if causes are known and unrelated to NP-C)
6. Pneumonia ☐1
   (should only be scored if pneumonia is likely related to NP-C, e.g. due to alveolar infiltrations or neurological impariment)
7. Apnea ☐1
   (as signs of brain stem damage or lung failure all apneas independent of neurological onset should be scored)

### 2.) Development

While initially most NP-C patients develop normally, a delay in reaching specific milestones is common. Developmental delay should be scored as follows:

1. Impaired psychomotor development at any time in postnatal/early infantile period
   (0-9 mo.) ☐1
2. Score 1P. for each milestone listed that is reached with delay (reference range in brackets). In case urine and/or feces continence are never reached, score 2P./symptom.

   Delayed milestone

   - Crawling (9-10 mo.) ☐1
   - Scrambling (9-10 mo.) ☐1
   - Sitting with and without support (6-10 mo.) ☐1
   - Attended Walking (12-18 mo.) ☐1
   - Free walking (18-24 mo.) ☐1
   - Grip (≈5 mo.) ☐1

- 1st words (12-18 mo.) ☐1
- urine continence acquired (<5years) ☐1 no☐2
- feces continence acquired (<5years) ☐1 no☐2

**3.) Motor functions**

In many NP-C patients decline of motor functions can be followed over many years. Accurate documentation of motor capabilities may therefore well reflect disease status. Each of the following motor signs is awarded 1P. or 2P. so that when summed up a maximum of 19 points may be reached in a completely immobile patient.

*Dysmetric symptoms*

1. Impaired fine motor skills ☐1
2. Impaired gross motor skills ☐2
(Impaired gross motor skills are scored higher due to greater impact on disease severity)

*Ataxic symptoms*

3. Balance problems ☐2
(as reported or clinically observed; note that balance problems may occur in patients that still have a normal gait)
4. Truncal ataxia ☐1
(difficulty maintaining upright posture, e.g. while sitting)
5. Unsteady gait ☐2
(gait ataxia as defined by abnormal clinical gait tests
6. Dydiadochokinesia ☐1
(inability to perform fast alternating movement of hands and/or feet)

*Deterioration of mobility*

7. running not possible ☐1
8. climbing/descending stairs not possible ☐1
9. standing alone not possible ☐2
10. walking aid needed ☐1
(this symptom is awarded a higher score than "standing with support not possible" as it represents decisive step during deterioration)
11. wheel chair bound ☐2
(scored if wheel-chair is needed for longer range mobility; patient may still be able to stand alone or to take a few steps on own feet)
12. standing with support not possible ☐1
13. no independent movement possible ☐3
(this symtom is awarded a high score due to its severe consequences)

*Other motor symptoms*

14. Dystonia ☐1
("Dystonia" includes hyperkinesia, athetosis, ballismus and chorea)
15. Tremor ☐1
The most important feature of tremor is that the rhythm is constant, producing a steady oscillation, although the amplitude of the movement may vary. All three kinds of tremor: rest tremor, intention tremor and peduncular tremor (with severe wide-amplitude tremor) should be considered. Clearly medication-induced tremor (e.g. upon Miglustat) is not to be considered for scoring.
16. Rigor/Rigidity ☐1

Rigidity is defined as muscle groups in affected limbs are rigid, which produces stiffness of movement. This stiffness has been linked to the sensation experienced when bending a lead pipe. (Ref. Patton, Neurological differential diagnosis)

17. Spasticity
    a. *Spastic drop foot* ☐3
    b. *Spastic legs* ☐3+1=4
    c. *Tetraspasticity* ☐4+3=7

Spasticity is defined as an increased muscle tone due to injury of the central nervous system. Spasticity primarily affects the limbs, starting with spastic drop feet (3P.). In a more advanced stage the whole leg may be spastic which affects the patient's ability to stand or walk (4P.). In the final stages of NP-C tetraspasticity may be present (7P.). Please note that for spastic drop feet 3P. are to be scored to which depending on the degree of impairment either 1.P (for spastic legs) or 4P. (for tetraspasticity) are to be added.

**4.) Ocular-motor abnormalities**

Over the course of disease, NP-C patients usually show worsening of characteristic ocular-motor abnormalities as a sign of increasing brain stem damage. Typical first ocular-motor signs are impaired voluntary saccadic eye movements (1P.), while slow eye pursuit is frequently unperturbed. Gaze palsy describes the complete paralysis of either looking up and down (vertical supranuclear gaze palsy, a rather specific NP-C sign; 2P.) or left and right (horizontal gaze palsy; 1P.). A patient with complete paralysis of voluntary eye movements in all directions is awarded all 4P. assigned to ocular-motor abnormalities.

1. Impaired saccadic eye movements ☐1
   (only voluntary saccades are impaired, slow eye pursuit normal)
2. Vertical gaze palsy ☐2
   (complete paralysis of looking up/down)
3. Horizontal gaze palsy ☐1
   (complete paralysisof looking left/right)

**5.) Seizures / cataplexy / narcolepsy**

1. Seizures
   Focal seizures ☐*4*
   Generalized seizures ☐ *(4+1=) 5*
   (may reflect brain damage. Focal seizures may arise by damage of only a distinct brain area, whereas generalized seizures (including absence seizures, myoclonic, and tonic-clonic seizures) typically involve larger brain areas. To reflect these different degrees of impairment, epilepsy that presents with focal seizures only is awarded 4P. Presentation with generalized seizures alone, or a mixture of focal and generalized seizures is awarded 5P.)
2. Cataplexy ☐2
   (is defined as episodes of sudden muscular weakness, which can cause the patient to collapse or fall. It can be triggered by emotion, especially laughter. The collapse is temporary and can range from sudden nods of the head to episodes involving the whole body.)
3. Narcolepsy ☐2
   (is much less common in NP-C than cataplexy, but may have severe impact on the daily and social life of a patient.)

**6.) Cognitive abilities and memory**

1. Cognitive impairment ☐3
   (Dementia and the loss of cognitive abilities in NP-C is gradual and frequently begins with learning problems in school. Some patient may have a cognitive impairment very early in their life)
2. Problems with short term memory ☐2

(Problems with short-term memory typically arise prior to problems with long-term memory which rarely manifests before disease state is progressed.)

3. Problems with long term memory ☐3
4. Supervision at home or at school needed ☐2

(after previous independence) Patients in a state requiring constant supervision (as a sign of progressed disease) are awarded additional 2P.

**7.) Behavioral and psychiatric abnormalities**

1. Psychomotor agitation/restlessness ☐1
   - constantly present/ in familiar environment ☐1

   (is defined as an inability to remain calm (relative to age group). 1P. is scored when this symptom only presents in an unfamiliar environment. An additional point is to be scored when this symptom is constantly present and/or occurs also in an environment is well known to the patient)
2. Social withdrawal ☐1

   (often occurs in patients who realize their inabilities and struggle with their disease. It has an impact on the patient's emotional life that may preceed other psychiatric problems.)
3. Apathy ☐2

   (is defined as a state of indifference. Emotions are suppressed, patients do neither show signs of happiness nor sadness.)
4. Moodiness ☐1

   (is defined as being liable to unpredictable changes of mood, especially sudden bouts of gloominess or sullenness.)
5. Sleeping disorder (dysomnia) ☐2

   (Patients with sleeping disorder (dysomnia) may have difficulties to fall asleep or to maintain sleep during the night.)
6. Frustration ☐1

   (is defined as the feeling of being upset or annoyed as a result of being unable to change or achieve something. In some NP-C patients, part of this frustration may turn into aggression towards themselves or others.)
7. Depression ☐1

   (as it its defined by the ICD10 includes major (depressive moods, lack of interest and happiness, increased tiredness, loss of energy) and minor symptoms (lack of concentration /attention, low self-esteem and suicidal thoughts).
8. Psychosis ☐3
   - Hallucinations/delusions ☐+1

   (refers to an abnormal condition of the mind that is characterized by impaired contact with reality. If psychosis manifests with hallucinations and/or delusions, additional 1P. is to be scored.)
9. Impaired vigilance ☐1

   (is defined as the inability to maintain attention and alertness over prolonged periods of time.)

**8.) Speech**

NP-C patients show deficits in the production as well as in the perception of speech.

1. Dysarthria ☐3
   - unitelligible speech ☐+2

   (means poor phonation and it is characterized by a slurred, irregular speech. Dysarthria usually starts with single words being inarticulate. If progressed to an almost unintelligible speech, additional 2P. should be awarded.)
2. Simplification of speech (decrease of vocabulary) ☐2

- Complete loss of speech ☐3

(is to be scored once a previously developed articulate speech starts to become simpler. Once a patient stops talking, additional 3P. are to be awarded.)

3. Dysphasia ☐2

(the loss of speech comprehension, is defined as the difficulty to understand written or spoken word and the inability to formulate appropriate answers, even though the patient is capable of phonation.)

4. Apraxia/Dyspraxia ☐2
   - Apraxia of daily tasks impaired (i.e. brushing hair) ☐1

Apraxia is defined as the loss of acquired skills, such as difficulty in simple household tasks (2P.). An additional 1P. is to be awarded if a patient is unable to perform a daily task such as brushing hair (when not due to motor dysfunction).

**9.) Hearing**

2. Hearing impairment ☐2

(is to be scored when auditive function has been objectively validated as reduced (if possible by technical measures such as audiometry, CERA, BERA).)

3. Complete deafness ☐3

is to be awarded an additional 3P.

**10.) Abilities in daily life**

1. Urine incontinence ☐3

and/or

2. Feces incontinence ☐3

mark a severe deterioration and are here to be scored **only if continence had been acquired** (see also “2. Development” for continence). Clearly medication-induced diarrhea and eventual feces incontinence (e.g. upon Miglustat) is not to be considered for scoring.

3. Washing ☐with aid 1 ☐not independent 2
4. Dressing/Undressing ☐with aid 1 ☐not independent 2

Loss of abilities of a patient to wash himself and to dress/undress are each to be scored with 1P. if the patient is still able to perform these tasks with aid/support and 2P. if the patient completely depends on others for these tasks.

5. Dysphagia ☐3
   - Aspiration of food ☐+1

(is a serious problem in NP-C patients as it may lead to aspiration. 1P. is scored additionally if in addition to fluids food cannot be swallowed properly.)

6. Drinking requiring aid ☐1

(e.g., straw) may reflect deterioration of dysphagia.

7. The need to be fed ☐3

in patients that have lost this ability typically reflects severe deterioration of independent lifestyle abilities.

8. Difficulties while feeding ☐1

(e.g., regurgitation, GI-Reflux) is to be scored by an additional point, as it is a critical threat for aspiration and pneumonia.

9. Gastric tubing ☐3

(whether past or present)
