# Supplementary figures and images for: Niemann-Pick disease type C clinical database: cognitive and coordination deficits are early disease indicators
Source: Orphanet J Rare Dis. 2013 Feb 22;8:35. doi: 10.1186/1750-1172-8-35 (PMC3649939; doi:10.1186/1750-1172-8-35)

Figure S1

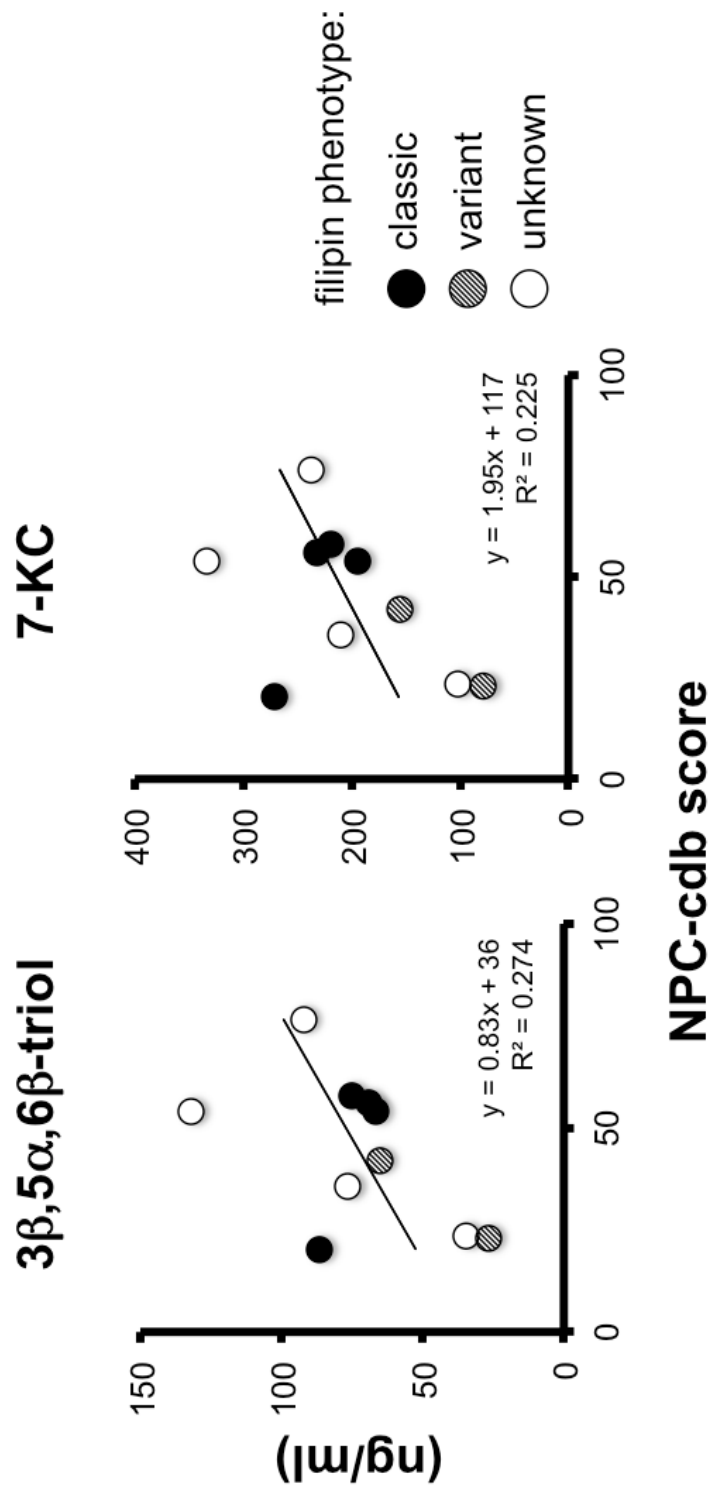

Supplement: Additional file 4: Figure S1 — Correlation of NPC-cdb score with plasma oxysterol levels and filipin phenotype. For 10 NP-C patients from our cohort, mean plasma levels (±SD) of cholestane-3β,5α,6β-triol (3β,5α,6β-triol) and 7-ketocholesterol (7-KC) were determined according to Jiang et al. [22] from a total of 19 samples (with a median of 2.5 samples per individual) (y-axis). Mean NPC-cdb scores per patient were generated from phenotypic information acquired at up to three consecutive visits (x-axis). Higher score values (y-axis) reflect higher number of symptoms. Where available, “classic” or “variant” filipin staining pattern are highlighted in grey. [file 1750-1172-8-35-S4.pdf]

**Figure S2**

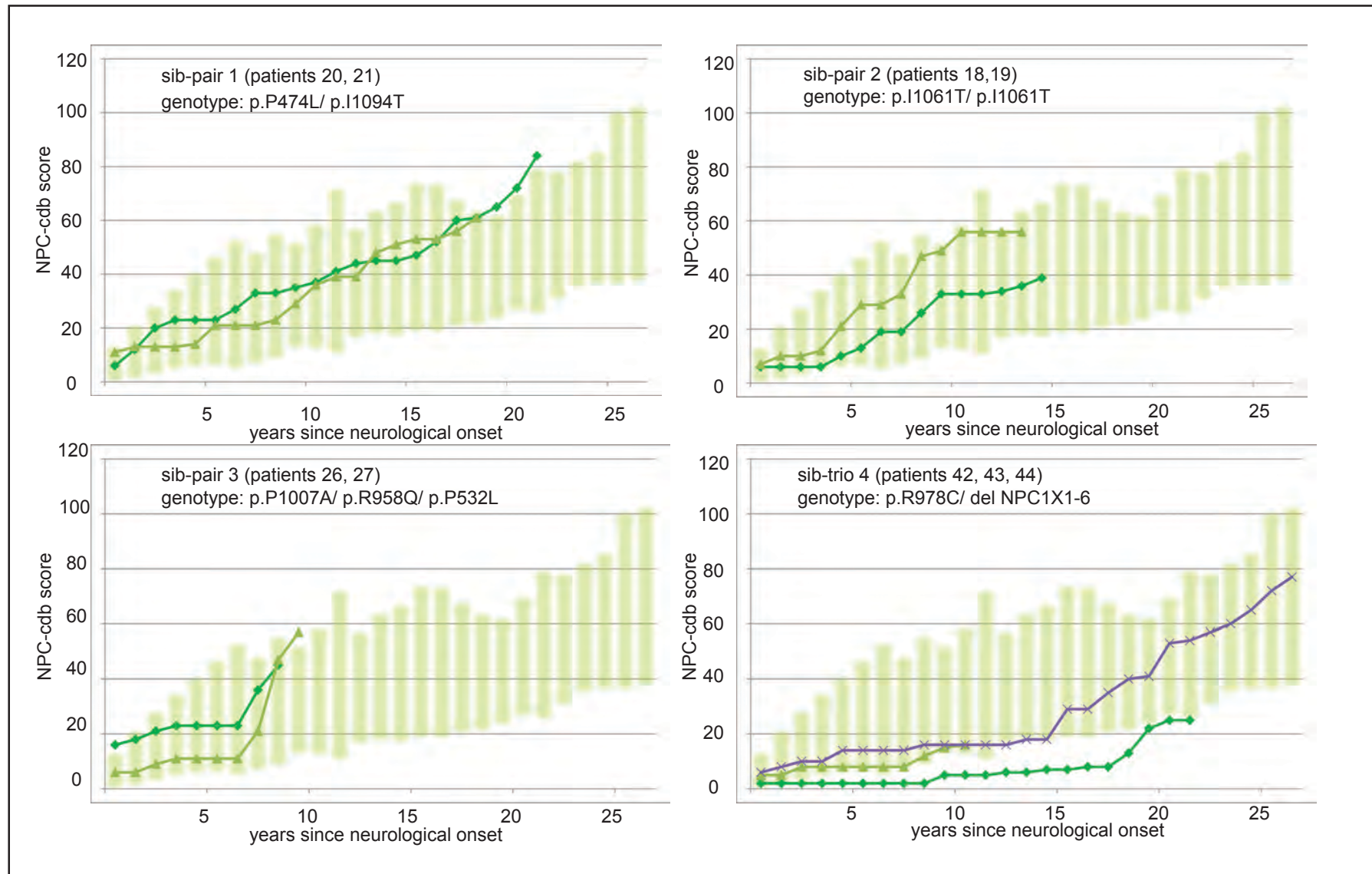

Supplement: Additional file 6: Figure S2 — Disease progression in siblings. Occurrence of disease symptoms over time (in years) was scored for each sibling of 3 NP-C sib pairs and one NP-C sib trio with the NPC-cdb score and normalized to the age at neurological disease onset (x=0). Higher score values (y-axis) reflect higher number of symptoms. Standard deviations (reflecting the normal range) are represented by shaded clouds. [file 1750-1172-8-35-S6.pdf]
