# Supplementary material for: Niemann-Pick disease type C clinical database: cognitive and coordination deficits are early disease indicators
Source: Orphanet J Rare Dis. 2013 Feb 22;8:35. doi: 10.1186/1750-1172-8-35 (PMC3649939; doi:10.1186/1750-1172-8-35)
Supplement: Additional file 5: Table S1 — Temporal development of 72 longitudinally-assessed NP-C symptoms (sorted acc. to frequency of occurrence). [file 1750-1172-8-35-S5.doc]

| **frequency (%)** | **no. of patients** | **symptom** | **censored with Kaplan-Meier method** | | | | | |
| --- | --- | --- | --- | --- | --- | --- | --- | --- |
|  |  |  | **mean**  **(years^1^)** | **95% CI of mean** | **stdv. mean** | **median (years^1^)** | **95% CI of median** | **stdv. median** |
| 86% | 36 | cognitive decline | 3.4 | (2.5-4.3) | 0.5 | 3 | (2.2-3.8) | 0.5 |
| 81% | 34 | starting dysarthria | 6.9 | (4.8-9.1) | 1.1 | 6 | (1.7-10.3) | 2.2 |
| 81% | 34 | clumsiness | 3.7 | (1.6-5.9) | 1.1 | 1 | (0.3-1.7) | 0.4 |
| 79% | 33 | ataxia | 7.5 | (5.5-9.5) | 1.0 | 6 | (4.7-7.4) | 0.7 |
| 79% | 33 | fine motor skills impaired | 4.8 | (2.5-7.1) | 1.2 | 2 | (0.8-3.2) | 0.6 |
| 76% | 32 | VSGP | 7.4 | (4.7-10.1) | 1.4 | 4 | (1.6-6.4) | 1.2 |
| 71% | 30 | severe dysarthria | 8.2 | (6.0-10.3) | 1.1 | 8 | (5.3-10.7) | 1.4 |
| 69% | 29 | dysphagia | 9.8 | (7.5-12.2) | 1.2 | 9 | (5.9-12.1) | 1.6 |
| 69% | 29 | unsteady gait | 8.9 | (5.9-11.9) | 1.6 | 6 | (2.1-9.9) | 2.0 |
| 64% | 27 | hand-feet-incoordination | 11.2 | (7.5-15.0) | 1.9 | 5 | (3.0-7.0) | 1.0 |
| 64% | 27 | balance problems | 9.2 | (5.5-12.9) | 1.9 | 6 | (3.4-8.6) | 1.3 |
| 62% | 26 | short-term memory impaired | 8.7 | (6.9-10.6) | 0.9 | 8 | (5.3-10.7) | 1.4 |
| 60% | 25 | gross motor skills impaired | 8.9 | (6.1-11.7) | 1.4 | 8 | (3.5-12.5) | 2.3 |
| 57% | 24 | saccadic eye-movement impaired | 12.8 | (9.7-15.9) | 1.6 | 10 | (7.8-12.2) | 1.1 |
| 55% | 23 | dysdiadochokinesia | 13.9 | (10.1-17.5) | 1.9 | 10 | (4.1-15.9) | 3.0 |
| 52% | 22 | spasticity | 14.1 | (10.5-17.7) | 1.8 | 13 | (8.6-17.4) | 2.2 |
| 48% | 20 | urine incontinence | 14.2 | (10.8-17.6) | 1.7 | 13 | (9.8-16.3) | 1.7 |
| 45% | 19 | constant supervision needed | 14.5 | (10.5-18.5) | 2.0 | 12 | (8.0-16.0) | 2.0 |
| 45% | 19 | dystonia | 14.2 | (10.7-17.8) | 1.8 | 13 | (7.8-18.2) | 2.6 |
| 43% | 18 | motor agitation | 15.7 | (11.4-20.0) | 2.2 | 16 | (5.0-27.0) | 5.6 |
| 43% | 18 | speech comprehension impaired | 16.1 | (12.0-20.2) | 2.1 | 13 | (8.5-17.5) | 2.3 |
| 43% | 18 | stool incontinence | 15.5 | (12.2-18.7) | 1.7 | 15 | (10.3-19.7) | 2.4 |
| 36% | 15 | seizures | 17.7 | (13.5-21.9) | 2.2 | 18 | (2.7-33.3) | 7.8 |
| 36% | 15 | long-term memory impaired | 15.2 | (12.1-18.3) | 1.6 | 15 | (10.6-19.4) | 2.2 |
| 36% | 15 | sleeping disorder | 16.4 | (12.6-20.2) | 1.9 | 16 | (13.1-19.0) | 1.5 |
| 36% | 15 | social withdrawal | 14.9 | (10.3-18.8) | 2.0 | 12 | na | na |
| 36% | 15 | tremor | 18.1 | (14.0-22.2) | 2.1 | 18 | na | na |
| 33% | 14 | frustration | 17.2 | (12.8-21.5) | 2.2 | 14 | (9.7-18.3) | 2.2 |
| 33% | 14 | horizonal saccades present | 19.1 | (15.0-23.2) | 2.1 | na | na | na |
| 33% | 14 | loss of speech | 16.6 | (13.1-20.0) | 1.8 | 17 | (10.1-23.9) | 3.5 |
| 31% | 13 | diarrhea | 18.8 | (14.7-22.8) | 2.1 | 19 | (12.9-25.1) | 3.1 |
| 31% | 13 | moodiness | 17.8 | (13.5-22.1) | 2.2 | 15 | (9.7-20.3) | 2.7 |
| 29% | 12 | apraxia | 18.7 | (14.6-22.8) | 2.1 | 20 | (13.1-26.9) | 3.5 |
| 29% | 12 | depressions | 17.6 | (12.9-22.2) | 2.4 | 18 | (10.8-25.2) | 3.7 |
| 29% | 12 | cataplexy | 20.6 | (16.7-24.5) | 2.0 | na | na | na |
| 29% | 12 | psychosis | 17.4 | (12.9-21.8) | 2.3 | 14 | (5.7-22.3) | 4.3 |
| 29% | 12 | running impossible | 18.5 | (13.4-23.5) | 2.6 | 17 | (6.7-27.3) | 5.3 |
| 26% | 11 | wheel-chair bound | 19.1 | (14.6-23.6) | 2.3 | 15 | na | na |
| 26% | 11 | spastic drop foot | 19.6 | (15.4-23.8) | 2.1 | 24 | (7.7-40.2) | 8.3 |
| 26% | 11 | starting loss of speech | 19.1 | (15.0-23.2) | 2.1 | 25 | (11.8-38.2) | 6.7 |
| 26% | 11 | delusions | 22.2 | (18.6-25.8) | 1.8 | 26 | (0.8-51.2) | 12.9 |
| 24% | 10 | absences | 21.4 | (17.5-25.3) | 2.0 | na | na | na |
| 24% | 10 | walking aid needed | 19.3 | (15.1-23.6) | 2.2 | 17 | (7.0-27.0) | 5.1 |
| 24% | 10 | hearing impairment | 18.7 | (15.1-22.4) | 1.9 | na | na | na |
| 24% | 10 | vigiliance disorder | 19.1 | (14.5-23.6) | 2.3 | 19 | (10.4-27.6) | 4.4 |
| 21% | 9 | feeding necessary | 20.8 | (16.5-25.0) | 2.2 | na | na | na |
| 21% | 9 | climbing stairs impossible | 20.8 | (16.6-25.0) | 2.1 | 20 | na | na |
| 21% | 9 | diapers needed | 22.1 | (18.1-26.0) | 2.0 | na | na | na |
| 19% | 8 | standing unassisted impossible | 21.6 | (17.3-26.0) | 2.2 | na | na | na |
| 17% | 7 | apathia | 22.5 | (18.2-26.7) | 2.1 | na | na | na |
| 17% | 7 | halluzinations present | 21.8 | (17.4-26.2) | 2.3 | na | na | na |
| 17% | 7 | grand-mal seizure | 23.3 | (19.5-27.0) | 1.9 | na | na | na |
| 17% | 7 | no independent movement possible | 22.3 | (18.0-26.5) | 2.2 | na | na | na |
| 17% | 7 | spasticity of the legs | 22.7 | (18.8-26.6) | 2.0 | 24 | na | na |
| 14% | 6 | dressing impossible | 23.6 | (20.1-27.0) | 1.8 | 26 | (20.0-32.0) | 3.1 |
| 14% | 6 | artificial nutrition needed | 23.0 | (19.1-26.9) | 2.0 | na | na | na |
| 14% | 6 | pneumonia | 22.5 | (18.4-26.6) | 2.1 | 24 | na | na |
| 14% | 6 | tetraspasticity | 22.9 | (19.2-26.7) | 1.9 | 24 | (19.7-28.3) | 2.2 |
| 14% | 6 | personal hygiene with assistance | 23.7 | 20.2-27.1) | 1.8 | 26 | (17.4-34.6) | 4.4 |
| 12% | 5 | muscular hypotonia | 25.5 | (22.7-28.4) | 1.5 | na | na | na |
| 10% | 4 | apnea | 24.3 | (20.3-28.4) | 2.1 | na | na | na |
| 10% | 4 | standing with assistance | 24.5 | (20.6-28.4) | 2.0 | na | na | na |
| 10% | 4 | focal seizures | 26.1 | (23.4-28.8) | 1.4 | na | na | na |
| 10% | 4 | rump ataxia | 24.4 | (20.4-28.5) | 2.1 | na | na | na |
| 5% | 2 | breathing therapy | 26.9 | (24.0-29.8) | 1.5 | na | na | na |
| 5% | 2 | (muscular) atrophy | 26.9 | (24.4-29.4 | 1.3 | na | na | na |
| 5% | 2 | febrile convulsions | 27.6 | 25.6-29.5) | 1.0 | na | na | na |
| 5% | 2 | rigor | 27.3 | (25.0-29.6) | 1.2 | na | na | na |
| 2% | 1 | autistic features | 28.3 | (26.9-29.7) | 0.7 | na | na | na |
| 2% | 1 | migraine | 28.3 | (27.0-29.7) | 0.7 | na | na | na |
| 2% | 1 | narcolepsy | 27.9 | (25.9-30.0) | 1.0 | na | na | na |
| 2% | 1 | constipation | 28.3 | (27.0-30.0) | 0.7 | na | na | na |

**Table S1 Temporal development of 72 longitudinally-assessed NP-C symptoms (sorted acc. to frequency of occurrence)**
